# New Appliances and Things Medical

**Published:** 1904-02-06

**Authors:** 


### NEW APPLIANCES AND THINGS MEDICAL.

[We shall be glad to receive, at our Office, 28 & 29 Southampton Street Strand, London, W.C., from the manufacturers, specimens of all new preparations and appliances which may be brought out from time to time.]

### ROYAL DOUBLE-CREAM CHEDDAR CHEESE.

(THE SOMERSET, DORSET, AND DEVON DAIRY COMPANY, LIMITED, CHARD, SOMERSET.)

IT is often said that cheese digests everything but itself, a remark which implies popular recognition of the fact that most kinds of cheese are comparatively indigestible, a circumstance which compels many individuals to exclude the article from their everyday dietary. It is with pleasure, therefore, that we are able to recommend the above cheese on the ground that it is an exception to the general rule, and is very readily assimilated. Containing as it does large percentages of fat and proteid it is highly nutritious, and it is likely, therefore, to be of especial value to convalescents and chronic invalids who require to take an abundance of easily digested food. Moreover, its mild and very pleasant flavour will make this cheese a welcome addition to every table.

